# Apoptotic Cells Induced Signaling for Immune Homeostasis in Macrophages and Dendritic Cells

**DOI:** 10.3389/fimmu.2017.01356

**Published:** 2017-10-25

**Authors:** Uriel Trahtemberg, Dror Mevorach

**Affiliations:** ^1^General Intensive Care Unit, Hadassah-Hebrew University Medical Center, Jerusalem, Israel; ^2^Rheumatology Research Center, Department of Medicine, Hadassah-Hebrew University Medical Center, Jerusalem, Israel

**Keywords:** clearance of apoptotic cells, toll-like receptor, nuclear factor kappa B, inflammasome, lipid-activated nuclear receptors, TAM receptors, signal transducer and activator of transcription 1, suppressor of cytokine signaling

## Abstract

Inefficient and abnormal clearance of apoptotic cells (efferocytosis) contributes to systemic autoimmune disease in humans and mice, and inefficient chromosomal DNA degradation by DNAse II leads to systemic polyarthritis and a cytokine storm. By contrast, efficient clearance allows immune homeostasis, generally leads to a non-inflammatory state for both macrophages and dendritic cells (DCs), and contributes to maintenance of peripheral tolerance. As many as 3 × 10^8^ cells undergo apoptosis every hour in our bodies, and one of the primary “eat me” signals expressed by apoptotic cells is phosphatidylserine (PtdSer). Apoptotic cells themselves are major contributors to the “anti-inflammatory” nature of the engulfment process, some by secreting thrombospondin-1 (TSP-1) or adenosine monophosphate and possibly other immune modulating “calm-down” signals that interact with macrophages and DCs. Apoptotic cells also produce “find me” and “tolerate me” signals to attract and immune modulate macrophages and DCs that express specific receptors for some of these signals. Neither macrophages nor DCs are uniform, and each cell type may variably express membrane proteins that function as receptors for PtdSer or for opsonins like complement or opsonins that bind to PtdSer, such as protein S and growth arrest-specific 6. Macrophages and DCs also express scavenger receptors, CD36, and integrins that function *via* bridging molecules such as TSP-1 or milk fat globule-EGF factor 8 protein and that differentially engage in various multi-ligand interactions between apoptotic cells and phagocytes. In this review, we describe the anti-inflammatory and pro-homeostatic nature of apoptotic cell interaction with the immune system. We do not review some forms of immunogenic cell death. We summarize the known apoptotic cell signaling events in macrophages and DCs that are related to toll-like receptors, nuclear factor kappa B, inflammasome, the lipid-activated nuclear receptors, Tyro3, Axl, and Mertk receptors, as well as induction of signal transducer and activator of transcription 1 and suppressor of cytokine signaling that lead to immune system silencing and DC tolerance. These properties of apoptotic cells are the mechanisms that enable their successful use as therapeutic modalities in mice and humans in various autoimmune diseases, organ transplantation, graft-versus-host disease, and sepsis.

## Introduction

Inefficient clearance of apoptotic cells contributes to systemic autoimmune disease in humans and mice (1–6), and inefficient chromosomal DNA degradation by DNAse II leads to systemic polyarthritis and a cytokine storm (7, 8). By contrast, efficient clearance allows immune homeostasis, generally leads to a non-inflammatory state for both macrophages and dendritic cells (DCs), and contributes to maintenance of peripheral tolerance (9–12).

As many as 3 × 10^8^ cells undergo apoptosis every hour in our bodies (13), where they are engulfed by macrophages and immature DCs and possibly by neighboring cells. What are the signaling patterns that induce these non-inflammatory responses?

Apoptotic cells characteristically expose “eat me” signals to macrophages (14, 15), and one of the primary “eat me” signals is phosphatidylserine (PtdSer). PtdSer is a phospholipid that localizes to the inner leaflet of plasma membranes in viable cells; however, when cells undergo apoptosis, it is exposed on the outer cell surface in a caspase-dependent manner (16–18). Other “eat me” signals exist and make important contributions to the process, including calreticulin from the dying cell endoplasmic reticulum and externally exposed chromatin and DNA, as well as alterations in surface charge and changes in glycosyl groups (19–21).

Apoptotic cells also produce “find me” signals to attract macrophages (22). Lysophosphatidylcholine (LPC) (22), fractalkine (23), sphingosine-1-phosphate (S1P) (24), and ATP/UTP (25) are released from apoptotic cells in a caspase-dependent manner. They also contribute to the “anti-inflammatory” nature of the engulfment process by secreting “tolerate me” signals *via* thrombospondin-1 (TSP-1) secretion (26) or “calm-down” signals *via* adenosine monophosphate (AMP) (27) and possibly other immune modulation signals yet to be discovered.

Another mechanism for immune modulation by apoptotic cells involves the caspase-dependent oxidation and deactivation of deactivation of high mobility group box 1 (HMGB1), a strong trigger of danger-associated-molecular-pattern (DAMP) that causes inflammatory responses (28). Similarly, in the context of viral infection, caspases can modify the mitochondria-initiated cell death process and inhibit the interferon (IFN) response, switching the result of the dying process from pro-inflammatory to immunologically silent (29, 30). Since the activation of caspases is not a necessary condition for apoptosis, it could be that caspase activation, which drives the apoptotic program toward tolerogenic consequences, is another way that apoptotic cells “instruct” the cells clearing them regarding the nature of their death (31).

Neither macrophage subpopulations or DCs are “uniform” and each cell type may variably express membrane proteins that function as receptors for PtdSer (Tim-4, stabilin 2, and BAI1), or for opsonins that bind to PtdSer, milk fat globule-EGF factor 8 protein (MFGE8), ProS, and growth arrest-specific 6 (GAS6) (14). Masking the PtdSer on apoptotic cells prevents their engulfment by macrophages and induces autoantibodies (4) and inflammation (32), supporting the idea that PtdSer is not only an important “eat me” signal but also a “tolerate me” signal. Macrophages also express integrins that function *via* bridging molecules such as TSP-1, MFGE8, and complement (2, 9, 33). These integrins can contribute to both phagocytosis and inhibition of a pro-inflammatory immune response, for example, by scavenger receptor (ScR) SCARF1 (34), the immunoglobulin superfamily member leukocyte-associated Ig-like receptor 1 (CD305) (35), CD11b or CD11c (2, 9, 36), other ScRs, CD36, and possibly additional receptors that are important in multi-ligand interactions between apoptotic cells and phagocytes (2, 19, 26, 37). In addition, “cross-talk” exists and, for example, C1q-dependent induction of opsonins Gas6 and Protein S has been described (38, 39).

Macrophages express specific receptors for some of these “find me” signals (CX3CR1 for fractalkine, aS1PR1 for S1P, and P2Y_2_ for ATP and UTP), which may mediate migration to the dying cells (15). The “find me” signals are thought to prime macrophages for engulfment, as best exemplified by the enhanced expression of MFGE8 (40). On the other hand, some “find me” signals, for example, LPC, ATP/UTP, and S1P, may cause inflammation (41–43), contradicting the anti-inflammatory nature of the apoptotic process. How is the anti-inflammatory character of the apoptotic process maintained during cell death and engulfment? We will discuss several signaling patterns that have been identified.

Other modes of cell death that are immunogenic (44), including accidental cell death (necrosis), necroptosis, pyroptosis, and NETosis (45), will not be discussed here.

## Signaling Inhibition of Toll-Like Receptors (TLRs), Nuclear Factor Kappa B (NF-κB), and the Inflammasome

### Toll-Like Receptors

Toll-like receptors are membrane-associated innate immune sensors that recognize conserved microbial-associated molecular structures of invading pathogens. A classic example is lipopolysaccharide (LPS), which is expressed by Gram-negative bacteria that binds to TLR4 and induces pathogen-associated molecular patterns (PAMPs). What is mostly relevant to this review is that TLRs also detect host-derived, danger-associated molecular patterns (DAMPs) and alarmins that can be produced during immunogenic programmed cell death or cell necrosis, including HMGB1 and endogenous RNA and DNA that are normally hidden in TLR-inaccessible compartments but become exposed and are released during cell stress, inflammation, infection, or non-apoptotic death (46–48). TLRs are expressed by macrophages and DCs that are specifically important for interaction with apoptotic cells, but they are also expressed by natural killer (NK) cells, mast cells, and T- and B-lymphocytes, as well as by some non-immune cells, such as epithelial and endothelial cells (46–48).

Importantly, apoptotic cells downregulate the response to TLR receptors on both macrophages and DCs (9, 49–51). For ligand detection and co-receptor interactions, TLRs contain an ectodomain with multiple leucine-rich repeat domains involved in a portion of the transmembrane, and an intracellular toll/IL-1 receptor (IL-1R) homology domain (TIR) essential for signaling (46–48).

Toll-like receptors are expressed on the plasma membrane (e.g., TLR1, TLR5, TLR6, and TLR10), in intracellular endosomes (e.g., TLR3, TLR7–9, and TLR11), or in both compartments (e.g., TLR2 and TLR4) (52), and TLR localization critically regulates its signaling where the initial step following binding is recruitment of adaptor proteins. The two main known pathways are myeloid differentiation primary response gene 88 (MyD88)- and TIR-domain-containing adapter inducing interferon β (TRIF)-dependent. All TLRs except TLR3 use the MyD88-dependent pathway to initiate signaling, whereas TLR3 and TLR4 use the TRIF-dependent pathway to elicit induction of both pro-inflammatory cytokines and type I IFNs (52).

After recruitment to TLRs, MyD88 molecules cluster and recruit interleukin-1 (IL-1) receptor-associated kinases (IRAKs) through homotypic death–death domain interactions (53). An alternatively spliced form of MyD88 (MyD88s) lacks a short linker sequence between the death- and TIR domains. It binds to the TIR domain of TLRs but fails to recruit IRAK1, thereby inhibiting signaling (54).

Expression of other regulatory kinases within the IRAK family is induced following TLR signaling. Increased expression of IRAK-M or alternatively spliced variants of IRAK1 (e.g., IRAK1c) suppresses TLR signaling (55, 56). IRAK-M was originally reported to prevent dissociation of IRAK and IRAK4 from MyD88, and to block engagement of TRAF6, inhibiting signaling (56); however, later studies demonstrated the ability of IRAK-M to engage a separate MEK kinase 3-dependent signaling pathway for NF-κB activation. This pathway leads to IκB-a phosphorylation, but not degradation, and controls a limited set of inflammatory cytokines and negative regulators [suppressor of cytokine signaling (SOCS) 1, SHIP-1, A20] whose expression is not controlled by mRNA stability (57). IRAK1c is an alternatively spliced form of IRAK that lacks a region encoded by exon 11 of the IRAK1 gene, resulting in a kinase-inactive form of IRAK (55, 56). IRAK1c can heterodimerize with IRAK, thereby fine-tuning the level of IRAK activity. Activator proteins engaged in MyD88- and TRIF-dependent signaling pathways also become phosphorylated, and these events play a critical role in TLR signaling.

Apoptotic cells were shown to downregulate TLR signaling events by us and by other groups, with effects on LPS-TLR4, zymosan-TLR2, and possibly other events (9, 37, 49, 50, 58, 59). Inhibition of TLR signaling after *in vivo* apoptotic cell administration has been clearly demonstrated in mouse models (36, 59, 60). Taken together, this strongly supports the impact of apoptotic cells on the inhibition of TLR signaling pathways in different innate immune cell subsets. On the other hand, in some abnormal conditions where pathogenic autoantibodies opsonize self-antigens and apoptotic debris, immune complexes are formed and bind to TLR 7 and TLR 9 and trigger the production of IFN-α, a hallmark of SLE (61, 62).

### Nuclear Factor Kappa B

Nuclear factor kappa B is a major transcription factor that has been implicated as a critical regulator of gene expression in the setting of inflammation. NF-κB is ubiquitously expressed and is activated by a wide variety of stimuli, including pro-inflammatory cytokines such as tumor necrosis factor-α (TNFα) and IL-1, bacterial- or viral-derived PAMPs, and various types of stress (63, 64).

The NF-κB family consists of five DNA-binding members. NF-κB1 is synthesized as p105 and is processed into a DNA-binding subunit, p50 (65). Likewise, NF-κB2 is produced as p100, which serves as a precursor for the active transcription factor p52. p50 and p52 form various combinations of heterodimers with RelA (p65), c-Rel, and RelB. These DNA-binding complexes target distinct sets of genes for transcriptional activation (66). In addition, RelA and c-Rel can also activate gene transcription as homodimers (67). By contrast, p50 homodimers, which lack transactivation function, can instead suppress NF-κB target gene expression, for example, in response to stimulation by LPS (68, 69).

Depending on the extracellular stimuli and the receptors engaged, NF-κB activation mechanisms can be broadly classified into canonical and non-canonical pathways (70). In the absence of extracellular stimuli, transcriptional activity of NF-κB transcription factors is normally kept in check by sequestration in the cytoplasm (71). Following activation, the transient transcriptional activity of NF-κB is maintained by several mechanisms to prevent inflammation-induced tissue damage or malignancy associated with chronic NF-κB activation (72). IκBa is induced in an NF-κB-dependent manner, which contributes to the termination of NF-κB signaling in a negative feedback loop (73). In addition, p100, which also serves as an IκB-like protein termed “IκBd,” plays a critical role in terminating NF-κB activity (71). Activation of the canonical NF-κB pathway depends on the IKK complex, which contains two catalytic subunits (IKKα and IKKβ) and a regulatory subunit NEMO/IKKc (74). Catalytically active IKKβ phosphorylates IκBα, signaling its ubiquitination and proteasomal degradation (75) in response to various stimuli, including TNF receptor 1, IL-1R, and TLRs. By contrast, non-canonical NF-κB pathway activation is mediated by IKKα, which phosphorylates p100. This is followed by partial p100 degradation to generate p52 in response to stimulation *via* certain TNFR family members such as B cell-activating factor receptor (BAFF-R), CD40R, and lymphotoxin-β receptor (76).

The ubiquitin-editing enzyme A20 complex is a negative regulator of canonical NF-κB signaling. Mice lacking A20 develop severe inflammation and cachexia, and die prematurely (77). These mice exhibit persistent NF-κB and IKK activation and severe systemic inflammation in response to TNFα and sublethal doses of LPS (77). Thus, A20 is an important negative feedback regulator of NF-κB required for immune homeostasis. Liberated NF-κB dimers from IκBα and IκBδ translocate to the nucleus and activate transcription of various genes involved in innate and adaptive immunity (78). Deletion of A20 in DC leads to the development of pathologies in mice similar to those seen in humans with inflammatory bowel disease (IBD) and SLE, including autoantibodies, splenomegaly, nephritis, colitis, and even ankylosing spondylitis (79–81). Similarly, humans who have been identified to have mutations in the *Tnfaip3* gene that encodes A20 show auto-inflammation (82, 83). Most important, apoptotic cell uptake by A20-deficient DC fails to inhibit pro-inflammatory cytokine production in response to LPS (79) and A20 expression is upregulated in small intestinal lamina propria CD103^+^ DC in response to apoptotic IEC (84). Furthermore, small intestinal lamina propria CD103^+^ DC induction of A20 in response to apoptotic cells (84) shuts down both apoptotic cell phagocytosis and inflammation, and thus may limit the supply of self-antigen and its presentation in an inflammatory context (85).

Lipopolysaccharide-induced cytokine production is mainly mediated by activation of NF-κB, MAPKs, and IRF-3, and by induction of a type I IFN-mediated, signal transducer and activator of transcription 1 (STAT1)-dependent autocrine loop. Our group has suggested that the mechanism for apoptotic cell inhibition of pro-inflammatory cytokines, as originally showed by Fadok et al. and Voll et al. (49, 58), is due to inhibition of TLR and NF-κB signaling (37, 59) and inflammasome for IL-1β (86) (see below). Inhibition of NF-κB by apoptotic cells has been shown by others (87, 88) and by our team (37, 59, 86), and it is suggested that nuclear migration of p65 is inhibited at the transcriptional or post-transcriptional level (37, 86). In addition, Mer receptor tyrosine kinase (RTK) (MerTK, see below) was also found to activate the phosphatidylinositol 3-kinase/AKT pathway, which negatively regulates NF-κB (89).

### Inflammasome

Inhibition of NF-κB could not explain very rapid inhibition of IL-1β secretion by apoptotic cells (37); thus, additional mechanisms remained unexplained.

IL-1β is a pro-inflammatory cytokine produced primarily by activated monocytes and macrophages that is involved in the regulation of immune responses as well as the pathogenesis of several acute and chronic inflammatory diseases. Release of IL-1β is mediated by a two-step process: first, transcriptional induction of pro-IL-1β, and then caspase 1-mediated cleavage for the generation and secretion of IL-1β (86). Inflammasomes are high-molecular-weight cytosolic complexes that mediate the activation of caspase 1 and therefore enable rapid secretion of IL-1β and IL-18, which already exist as pro-cytokines. There are many inflammasomes, and each is influenced by a unique pattern recognition receptor response. Two signals are typically involved in inflammasome pathways (90). Signal one involves recognition of PAMPs or DAMPs that interact with TLRs, thus inducing downstream production of pro-IL-1β. This is followed by signal two, which involves recognition of PAMPs or DAMPs made by cells such as uric acid or ATP *via* nucleotide-binding domain, leucine-rich-containing family (NLR) pyrin domain-containing-3 (NLRP3), which leads to caspase-1-dependent cleavage of pro-IL-1β to active IL-1β. Both PAMPs and DAMPs can be liberated by early insults. The consequence of inflammasome activation and IL-1β expression is the upregulation of adhesion molecules and chemokines, leading to neutrophil sequestration, mononuclear phagocyte recruitment, and T cell activation.

Apoptotic cells were shown by us to inhibit TLRs and the NF-κB pathway (37, 59). TLR triggering is important for enhanced transcription of pro-IL-1β and pro-IL-18, and is in fact needed for the effect, but is not sufficient for rapid IL-1β secretion. We were able to show that apoptotic cells inhibit secretion of activated IL-1β at both pre- and post-transcription levels, and have distinct inhibition effects on NF-κB and NLRP3 (86). The dextran sulfate sodium (DSS) colitis model is generally viewed as an epithelial damage model suited to investigate innate immune responses. Macrophages primed with LPS and subsequently exposed to DSS secrete high levels of IL-1β in an NLRP3-, ASC-, and caspase-1-dependent manner. The DSS murine model and *Nlrp3*-deficient mice were used by us to assess the effect apoptotic cells on colitis. Immunohistochemistry, flow-cytometry, and Western blots helped to explore the effect and mechanisms. Using a variety of NLRP3 triggering mechanisms, we showed that apoptotic cells negatively regulate NF-κB and NLRP3 activation at the pre- and post-transcription levels *via* inhibition of reactive oxygen species (ROS), lysosomal stabilization, and blocking potassium efflux. This property of apoptotic cells was associated with a dramatic clinical, histological, and immunological amelioration of DSS colitis in Balb/c and B6 mice following a single administration of apoptotic cells (86).

Apart from apoptotic cell opsonization, MFGE8 was also found to be an endogenous inhibitor of inflammasome-induced IL-1β production (91). MFGE8 inhibited necrotic cell-induced and ATP-dependent IL-1β production by macrophages through mediation of integrin β3 and P2X7 receptor interactions in primed cells. *itgb3* deficiency in macrophages abrogated the inhibitory effect of MFGE8 on ATP-induced IL-1β production. Furthermore, in a setting of post-ischemic cerebral injury in mice, MFGE8 deficiency was associated with enhanced IL-1β production and larger infarct size. The latter was abolished after treatment with IL-1β receptor antagonist. MFGE8 supplementation significantly dampened caspase-1 activation and IL-1β production and reduced infarct size in wild-type (WT) mice, but did not limit cerebral necrosis in *IL-1*β*-, Itg*β*3-*, or *P2rx7*-deficient animals.

What is the mechanism by which apoptotic cells inhibit inflammasome? We could show the involvement of three mechanisms in the resolution by apoptotic cells of inflammasome-induced inflammation (86). First, other groups (92, 93) and our lab (86) have shown that apoptotic cells are able to reduce and inhibit the formation of ROS at rates similar to those shown for the chemical inhibitor *N*-acetyl cysteine. It is well established that macrophages make use of toxic ROS to control microbial pathogens as part of the innate immune response, and ROS were identified as major mediators of inflammatory signals believed to play a role in the development of IBD. Furthermore, generation of ROS was found to induce IL-1β *via* ERK phosphorylation (94). In addition, IL-1β signals may induce ROS generation (95). While it has been shown that DSS induces formation of ROS (96, 97), we were able to see a marked reduction in ROS generation, and consequently less IL-1β secretion, when macrophages were pretreated with apoptotic cells (86).

The second mechanism involves the lysosome. It was shown that lysosomal damage or leakage may serve as an endogenous danger signal and is sensed by the NLRP3 inflammasome (96, 98). We have analyzed involvement of the lysosome vacuole and were first to discover that lysosomes from peritoneal macrophages that were introduced to apoptotic cells were more stable to DSS challenge, and were not affected or damaged (86).

Inflammasomes were also suggested to be activated in response to signaling pathways that deplete intracellular potassium, such as the potassium ionophore nigericin pathway (99). We noted that when macrophages were pretreated with apoptotic cells, nigericin-induced IL-1β secretion was significantly inhibited. The mechanisms for apoptotic cell inhibition of this secretion are not clear.

Taken together, these results demonstrate a mechanism of inflammasome inhibition and resolution of inflammation stemming from apoptotic cell clearance and illustrate a mechanism for regulation of inflammation that could take place in both infectious and noninfectious inflammatory conditions.

Additional related possible mechanisms include a functional subgroup of NLRs that negatively regulate inflammation (100), including the possible effect of apoptotic cells on NLRP12. This suppressor of pro-inflammatory cytokine and chemokine production downstream of TLRs targets multiple points in the NF-κB pathway. However, it is clear that failure to clear apoptotic cells will trigger persistent inflammasome-dependent inflammation, as perhaps is seen in failure to clear intracellular organelles (101).

## Lipid-Activated Nuclear Receptors

Nuclear receptors are transcription factors that regulate gene transcription in response to their ligand and include lipids, vitamins, and hormones. They suppress or activate transcription, allowing regulation diverse biological functions that include cytokine production, lipid metabolism, and more (102). Following internalization of apoptotic cells, lipids, carbohydrates, protein, and nucleotides are acquired from the apoptotic cell. This content was suggested to be a significant metabolic burden on the phagocyte (103) and may also influence its immune response.

Several studies have shed light on the metabolic changes macrophages undergo to restore normal cellular function and homeostasis following apoptotic cell ingestion. Macrophages and DCs express several nuclear factors, most with a role in clearance of apoptotic cells, including peroxisome proliferator-activated receptor (PPAR)-α, -β/δ and -γ isotypes, liver x receptor (LXR) α and β isotypes, retinoid x receptor (RXR) α and β isotypes, retinoic acid receptor, vitamin D receptor, and glucocorticoid receptor (104). The putative natural ligands of LXR are oxysterols, which have been suggested to arise from the lipids derived from apoptotic cell/body membranes. This exemplifies the anti-inflammatory effects of the PPAR and LXR receptors (104).

Mukundan et al. (105) and A-Gonzalez et al. (106) showed how lipids in apoptotic cells induce activation of the transcription factors PPAR-δ and LXR, respectively. Activation of these transcription factors results in macrophage upregulation of cell surface receptors and soluble ligands that suppresses inflammatory cytokine production and promotes removal of apoptotic cells. In their absence, apoptotic cell clearance is impaired and a lupus-like autoimmunity develops.

Concerning the mechanism of action, like other members of the same nuclear receptor family, PPAR-δ and LXR heterodimerize with the RXR to regulate transcription of target genes. Depending on the presence of corepressors or coactivators, these dimeric transcription factors can either suppress or initiate transcription (107).

Mukundan et al. (105) showed that the lipids contained in apoptotic cells activate PPARs. They observed that PPAR-δ, but not PPAR-ϒ, was induced in macrophages after exposure to apoptotic cells. Furthermore, macrophages obtained from mice deficient in PPAR-δ showed reduced engulfment of apoptotic cells and impaired clearance of apoptotic cells *in vitro* and *in vivo*. To prove that these changes were due to defects in macrophages, they created mice that were PPAR-δ-deficient exclusively in macrophages and observed similar defects in clearance of apoptotic cells, but not of necrotic cells.

A-Gonzalez et al. (106) observed that knockout of the genes encoding both the α and β chains of LXR led to defective phagocytosis of apoptotic cells. They then identified candidate genes responsible for this defect by microarray. In contrast to the reduced expression of opsonins observed with PPAR-δ deficiency, loss of LXR led to a marked reduction in the expression of the macrophage receptor Mer (see below), which binds either the GAS6 or Pro S opsonins. Both of these opsonins attach to PtdSer on apoptotic cells, resulting in apoptotic cell engulfment. Experimental support for regulation of Mer by LXR was obtained by detection of LXR binding to the promoter of *Mertk* (which encodes Mer) by gain of function experiments and by demonstration of increased phagocytosis of apoptotic cells after macrophage stimulation with the synthetic LXR agonist GW3965. The findings suggest that, after the ingestion of apoptotic cells, oxysterols (oxidized derivatives of cholesterol) activate transcription of Mer by LXR and like PPAR-δ. This transcription factor enhances the clearance of dying cells (104).

Macrophage activation *via* adenosine receptors is followed by the upregulation of TSP-1 and Nr4a gene expression, especially of *Nur77*, NOR-1, and *Nurr1*. TSP-1 is the major activator of TGFβ (108) and Nr4a family members inhibit the expression of pro-inflammatory cytokines such as TNFα, IL-8, and IL-6 in macrophages (109) by recruiting a repressor complex to their promoter (110). Yamaguchi et al. (27) found that AMP was present at 30- to-100-fold higher concentrations than ATP in the culture supernatants of apoptotic thymocytes and a T cell line. When cells undergo apoptosis, ATP is quickly hydrolyzed to AMP, while its generating system is inactivated by caspases (111). The caspase-cleaved Pannexin channel also contributes to cellular loss of ATP by allowing ATP to exit cells through the plasma membrane. Chekeni et al. (112) showed that caspases cleave Pannexin 1 in the early stages of apoptosis, resulting in the release of ATP, which can serve as a “find me” signal to attract macrophages. Depending on the types of cells and apoptotic stimuli, intracellular ATP levels remain high (113–115) or rapidly decrease (116, 117).

## Inhibition of Inflammation by *Tyro3, Axl*, and *Mertk* (TAM) Receptors

Early indications that these RTKs may have an important immune function came from observations made in studies by Camenisch et al. (118), who used *Mertk*^−/−^ mice. These mice had an approximately threefold enhancement of serum TNFα when administered 100 mg/kg of LPS *in vivo*. In 2001, Lu and Lemke (119) further characterized RTK’s role in the immune system by using mice that were triple knockout for TAM receptors *Tyro3*^−/−^ *Axl*^−/−^ *Mertk*^−/−^ (TAM RTK). These TAM RTK animals did not present with serious developmental anomalies and appeared normal (119). They had apparently normal immune systems with no differences in the size of the secondary lymphoid organs, and their role in inflammation was only clarified later.

The TAM receptors were originally discovered by Lai and Lemke (120). TAM receptors are key inhibitors of the immune system (121). Diverse immune cells in humans and mice express TAM components and are severely perturbed if their TAM-dependent cellular pathways are ablated (122). TAM signaling provides an indispensable inhibitory feedback mechanism responsible for safeguarding the shutdown of inflammation and promotion of tissue-repair processes. Blocking TAM signaling causes severe defects in apoptotic cell clearance, widespread inflammation, overactivation of the immune system, and development of systemic autoimmunity (123, 124).

TAM receptors are activated *via* two known mediators, ProS and GAS6 (125). Both bind to apoptotic cells and thereafter mediate ligation to TAM receptors. Both are Gla domain-containing proteins, i.e., proteins containing gamma-carboxylated glutamic acid residues. The gamma-carboxylation of glutamate residues vastly increases their ability to bind Ca^2+^. GAS6 and ProS contain Gla domains consisting of ~50 amino acids stretched near their N termini. Gamma-carboxylation and PtdSer binding are essential for the maximal bioactivity of both full-length TAM ligands (126–129). For example, ProS has a cofactor activity; its ability to activate the TAM RTKs is dependent on gamma-carboxylation of the GLA domain and binding to PtdSer (130).

The current thought is that TAM RTKs are significantly upregulated as part of a pro-inflammatory response, for example, TLR engagement. However, in the case of apoptotic cell clearance there may be direct signaling following receptor binding. For example, and as mentioned earlier, A-Gonzalez et al. (106) observed that knockout of the genes encoding both the alpha and beta chains of LXR led to a marked reduction in the expression of Mer. Experimental support for regulation of Mer by LXR was obtained by detection of LXR binding to the promoter of *Mertk*, which encodes Mer, by gain of function experiments and demonstration of increased phagocytosis of apoptotic cells after macrophage stimulation with the synthetic LXR agonist. The findings suggest that, after the ingestion of apoptotic cells, oxysterols (oxidized derivatives of cholesterol) activate transcription of Mer by LXR and like PPAR-δ.

## Inhibition of IFNs by SOCS 1/3 Upregulation

### Type II IFN

The phenotype that defines activated macrophages and DCs is characterized by increased microbicidal or autoimmune activity, high antigen-presenting activity associated with increased MHC class II expression, and increased production of IL-12 (131). These characteristics are promoted by an IFNγ-mediated Janus kinase signal transducer and activator of transcription (JAK-STAT) signaling. Stimulation of the IFNγ receptor triggers JAK-mediated tyrosine phosphorylation and subsequent dimerization of STAT1, which binds as a homodimer to elements known as gamma-activated sequences in the promoters of the genes encoding NOS2, the MHC class II transactivator, and IL-12, among others (132).

We were the first to show that clearance of apoptotic cells inhibits type II IFN (human γ-IFN) signaling by upregulation of SOCS (59). We showed that interaction of macrophages with apoptotic cells had no activation effect for MAPKs p38, JNK, or ERK1/2 (59). By contrast, apoptotic cells suppressed the LPS-induced IFN-mediated autocrine loop by attenuating STAT1 activation and suppressing IFN activation of STAT1-dependent genes such as CXCL10 (133, 134). It has been suggested that apoptotic cell induction of SOCS1 and SOCS3 expression contributes to suppression of IFN-induced gene expression (135), and thus suppresses JAK-STAT signaling and IFN-mediated responses downstream of TLR4.

Interferon-γ is a key activator of macrophages and is mainly produced by NK cells, Th1 cells at later stages of the immune response, and chimeric antigen receptor T cells. STAT1 mediates most of the IFN-γ activating effects on macrophages. We analyzed the effect of apoptotic cells on the IFN-γ signaling pathway in macrophages both *in vitro* and *in vivo*. We found that IFN-γ induced STAT1 activation at both the tyrosine phosphorylation and DNA-binding levels, and was significantly inhibited in macrophages that had interacted with apoptotic cells *in vitro* and *in vivo* in a chemically induced peritonitis murine model of inflammation. Inhibition of STAT activation was somewhat selective for STAT1 relative to STAT3, which is activated by IL-10 and is strongly anti-inflammatory. This selective inhibition pattern of cytokines and STATs would have the net effect of suppressing inflammatory macrophage activation while leaving deactivation pathways intact.

Suppressor of cytokine signaling ubiquitin ligases are responsible for downregulation of the immune response through the turnover of molecules that function in critical, positive, regulatory signaling cascades such as the TLR, NF-κB, and JAK-STAT pathways (136). Substrates of SOCS1 and SOCS3 include MAL, TRAFs, and JAKs.

### Type I IFN

Rothlin and Lemke showed later that the TAM RTK-dependent upregulation of SOCS required the type I IFN receptor and STAT1 (89). Consistent with a central role for TAM RTK in the negative regulation of inflammation, the upregulation of SOCS by type I IFNs was contingent on TAM RTK. SOCS1 induction by IFN-α was significantly reduced in TAM RTK TKO DCs (89). Axl mRNA was induced by type I IFNs produced downstream of TLR activation (89, 137, 138), indicating that the braking mechanism is not available at the onset of the immune response but only following its initiation.

The mechanism of TAM RTK action in type I IFNs involves upregulation of the SOCS proteins SOCS1 and SOCS3 (89). Consistent with a central role for TAM RTK in the negative regulation of inflammation, the upregulation of SOCS by type I IFNs was contingent on TAM RTK. SOCS1 induction by IFN-α was significantly reduced in TAM RTK TKO DCs (89). It was suggested that TAM RTKs can complex with type I IFN receptors and modify STAT function (89), potentially by altered phosphorylation. An additional mechanism of TAM RTK-mediated inhibition of inflammation includes upregulation of the transcription factor twist, which in turn leads to downregulation of TNFα (138).

Lipopolysaccharide-induced cytokine production is mainly mediated by activation of NF-κB, MAPKs, and IRF-3, and by induction of a type I IFN-mediated STAT1-dependent autocrine loop (139).

*Taken together*, these observations illustrate tight negative regulation of type I (89) and II (59) IFN pro-inflammatory signaling.

## Apoptotic Cells and Maintenance of Peripheral Tolerance

Recognition of an autoantigen by the T cell receptor (TCR) is the capability that distinguishes autoreactive T cells from other T cell subsets. The TCR repertoire is generated in immature T cells in a relatively random manner, and some TCRs recognize self-antigens. The majority of T cell clones with high-affinity TCRs that recognize self are deleted as a consequence of self-antigen presentation by thymic epithelial cells (140). Thymic selection is imperfect; therefore, autoreactive T cells are present in the peripheral T cell repertoire of healthy individuals (141). T cells that escape negative selection in the thymus must be held in check by additional peripheral tolerance mechanisms, and the ability to tightly control and avoid the activation of peripheral self-reactive T cells is crucial for avoiding autoimmunity.

Dendritic cells are the most potent antigen-presenting cells, and as such they are key regulators of the immune system (142). They share with macrophages many of the roles described earlier in the engulfment and clearance of apoptotic cells (85). Two main issues differentiating DCs from macrophages, in the context of this review, are the existence of more than two subpopulations of DCs with different roles and anatomical locations (85, 143), and their main role initiating the adaptive immune response classically described after migrating to a lymphoid organ (144). For example, CD8α^+^ murine DCs and their suggested human analog, the CD141^+^ DCs (145) are specialized in the uptake of apoptotic cells and the cross-presentation of their antigens to T cells (146). In this review, we will concentrate on myeloid DCs, although there are reports on the effects of plasmacytoid DCs after engulfment of apoptotic cells (147), and they can have anti-inflammatory effects *via* mechanisms similar to those used by myeloid DCs (148).

T cell activation requires a first signal provided by TCR ligation and a second signal provided by engagement of costimulatory molecules with their respective ligands on antigen-presenting cells. A third signal related to IL2/IL2R interactions should also sometimes be considered. The coordinated triggering of these two independent signaling systems ensures full T cell activation, including proliferation and acquisition of effector function. TCR occupancy in the absence of costimulatory signals leads to a sustained loss of antigen responsiveness called clonal anergy or T cell apoptosis, and therefore DCs interacting with apoptotic cells contribute to the maintenance of peripheral tolerance (9, 12, 149). We were able to show downregulation of costimulatory molecules, including CD40 and CD86 as well as MHC-DR, following apoptotic cell ingestion by DCs. Interestingly, DCs did not lose the ability to migrate to the lymph nodes following this effect, and upregulated CCR7, which is normally upregulated upon activation and allows tolerogenic DCs to migrate to naïve areas of the lymphatic system to encounter potential autoimmune T cells that may react to apoptotic cell antigens (9, 12).

Lung DCs are an excellent example of the dual role of DCs that can induce tolerance or activate naïve T cells, making these DCs well-suited to their role as lung sentinels. Lung DCs are supposed to serve as a functional signaling/sensing unit to maintain lung homeostasis and orchestrate host responses to benign and harmful foreign substances [reviewed in Ref. (150)]. *In vitro* observations demonstrating this role were further investigated using *in vivo* murine studies. CD11c+ CD11b+ CD8α+ DCs were found in the pancreatic lymph nodes, carrying fluorescently labeled dead cells that had been injected directly into the pancreas (151). Furthermore, apoptotic cells were phagocytosed by CD8α+ DCs within the spleen and apoptotic cell antigen was cross-presented to antigen-specific CD8 T cells, leading to their deletion as a mechanism of immune tolerance (10, 11).

Later, a role for specialized CD169+ macrophages in handling dead tumor cells and cross-presentation was also suggested (152), and identification of special subsets in different localizations within organs like lung, brain, and gut led to the suggestion that the nature of a phagocyte that recognizes, samples, and/or internalizes apoptotic cells is likely dependent on the tissue and its physiological state at any given time (84, 85). Further details are found elsewhere (85, 153).

With respect to apoptosis of antigen-presenting cells, we were able to show that apoptotic monocytes secrete TSP-1, which, by itself, induces a tolerogenic phenotype in DCs (26). While it has long been known that apoptotic cells change their phenotype during the death process (154), this has not been given much attention in the literature. Our group has shown that there are two subpopulations of human monocyte-derived DCs with different immune phenotypes and functions. Apoptotic cells are not necessarily homogeneous; thus, upon entering the process of apoptotic cell death, these two cell types differentially regulate their expression of cell surface antigens in a way that will dramatically influence interaction with T cells (149). Thus, even while dying, these DCs are explicitly signaling the cells they interact with and conveying information, their “immune will.” We identified three general patterns of expression: Pattern 1, surface marker expression increases for both subpopulations as cell death progresses; Pattern 2, surface marker expression increases in one subpopulation; and Pattern 3, surface marker expression shows a mixed pattern as cell death progresses with behavior dependent on the stimuli used. Importantly, one subpopulation dramatically increased CD86 expression in correlation to advanced apoptosis, suggesting that even during the death process DCs can signal to T cells for immune responses.

In another example, it has been shown that the susceptibility of different DC subpopulations to apoptosis has significance for the immune response to viral infections (155). These processes highlight the importance of apoptosis of the antigen-presenting cells themselves as an immune regulatory event that is a less recognized way for apoptosis to affect the immune system.

One of the main effects of DCs after the uptake of apoptotic cells is secretion of TGF-β, IL-10, and retinoic acid, which promote the development of T-regulatory (Treg) cells (85, 156–159).

These could be induced *via* the abovementioned mechanism, but also by the induction of indoleamine 2,3 dioxygenase-1 (IDO-1), whose activity can be induced by TGF-β (160) and whose expression is induced by apoptotic cells. Both *in vitro* (161, 162) and *in vivo* administration of apoptotic cells to mice induce the expression of IDO in splenic marginal zone macrophages (163). IDO-1 promotes immune tolerance by the induction of Treg cell differentiation (163–168).

Other studies have shown that DC phagocytosis of apoptotic cells initiates naïve CD4 T cell differentiation into Treg cells (169–174).

In conclusion, apoptotic cells induce a tolerogenic DC phenotype and may directly (anergy- or activation-induced cell death) or indirectly (Tregs) inactivate potential autoreactive T cells. In this way they represent a potent peripheral tolerance mechanism.

## Apoptotic Cells as Therapeutic Agents

The *in vitro* and *in vivo* properties of apoptotic cells suggest their potential use in a broad range of inflammatory and immune-mediated conditions such as autoimmunity, graft rejection, post-ischemic injury, cytokine storm, and more.

Autoimmune and autoinflammatory conditions, including type 1 diabetes in non-obese diabetic mice, experimental autoimmune encephalomyelitis, arthritis, colitis, pulmonary fibrosis, fulminant hepatitis, contact hypersensitivity, acute- and chronic-graft rejection, hematopoietic cell engraftment, acute graft-versus-host disease (GvHD), and reduction of infarct size after acute myocardial infarction have all been treated quite successfully by apoptotic cell infusion (175). While these works have been performed in animal models, our group has shown a remarkable reduction in the occurrence of grade II–IV GvHD following heterologous hematopoietic stem cell transplantation in humans with apoptotic cell treatment (176).

In the IBD study mentioned earlier (86), a single infusion of apoptotic cells significantly ameliorated both the clinical score and histological appearance of DSS-induced colitis. We showed that apoptotic cell infusion is beneficial in murine models of IBD and inhibits both inflammasome- and NF-κB-dependent inflammation.

In another example of the use of apoptotic cells for the treatment of inflammatory conditions in post-ischemic cerebral injury in mice, MFGE8 deficiency was associated with enhanced IL-1β production and larger infarct size. The latter was abolished after treatment with IL-1β receptor antagonist. MFGE8 supplementation significantly dampened caspase-1 activation and IL-1β production and reduced infarct size in WT mice, but did not limit cerebral necrosis in *IL-1*β*-, Itg*β*3*-, or *P2rx7*-deficient animals (91).

Lipopolysaccharide is a main causative agent of Gram-negative bacterial septic shock. Ren et al. (177) examined the possible protective effect of apoptotic cell infusion. They found that when apoptotic cells were administered 24 h after LPS challenge, B6 mice benefited, with a reduction in circulating pro-inflammatory cytokines, suppression of polymorphonuclear neutrophil infiltration in target organs, decreased serum LPS levels, and decreased mortality. Interestingly, LPS can quickly bind to apoptotic cells and these LPS-coated apoptotic cells can be recognized and cleared by macrophages accompanied with suppression of TNFα and enhancement of IL-10 expression. LPS-treated mice began to die at 8–12 h and all mice died within 3 days in the control group. By contrast, mice in the group injected intravenously (IV) with apoptotic cells (1 × 10^7^/mouse) immediately after challenge with LPS exhibited fewer signs of sickness. Only 20% of treated mice died at day 7; that is, treatment with apoptotic cells resulted in 80% survival (*n* = 12, *p* < 0.001). Late deaths in the treatment group were not observed during the 3 weeks after LPS injection, indicating that apoptotic cell treatment conferred a complete and lasting protection against lethal endotoxemia.

To further examine whether the administration of apoptotic cells has a beneficial effect in another animal model, the authors induced sepsis in mice by cecal ligation and puncture (CLP) (177). Without any treatment, 52% of the mice (12 of 25) died within 5 days. Mice with apoptotic cell treatment 1 h after CLP exhibited fewer signs of sickness and less than 20% of treated mice (4 of 22) died in the first 3 days. The authors also investigated whether delayed administration of apoptotic cells would prevent mice from endotoxic lethality. Treatment with apoptotic cells was initiated 1, 3, 6, and 24 h, respectively, after the onset of endotoxemia. Delayed treatments at all time points significantly protected mice from lethal shock (*n* = 6/group, *p* < 0.05). No late deaths occurred during the subsequent 3-week period of observation. These results indicated that delayed administration of apoptotic cells in mice provided protection from LPS-induced lethal shock.

Other works showed that timing is important, and if apoptotic cells were given 5 days before sepsis induction, worsened survival was observed (178). Sepsis and septic conditions were also examined *in vivo* in *Mertk*^−/−^ mice, by Camenisch et al. (118), who used *Mertk*^−/−^ mice. *In vivo*, the LD_50_ of LPS for *Mertk*^−/−^ mice was half of that for WT mice. *Mertk*^−/−^ mice had an approximately threefold enhancement of serum TNFα when administered 100 mg/kg of LPS, and about 90% of mice died of endotoxic shock (118).

Taken together, these studies suggest that the best timing for apoptotic cell treatment during sepsis is after its onset. The treatment response mechanism is most probably a systemic increase in the ability to return to a homeostatic state and a reduction of the intensity of the initial unwanted immune response characterized by cytokine storm. Other possible mechanisms by which apoptotic cells could provide protection in sepsis include binding to toxins, promoting APC survival, and recovering APCs from their septic “reprogramming” (179, 180).

## Summary

**Figure 1:**
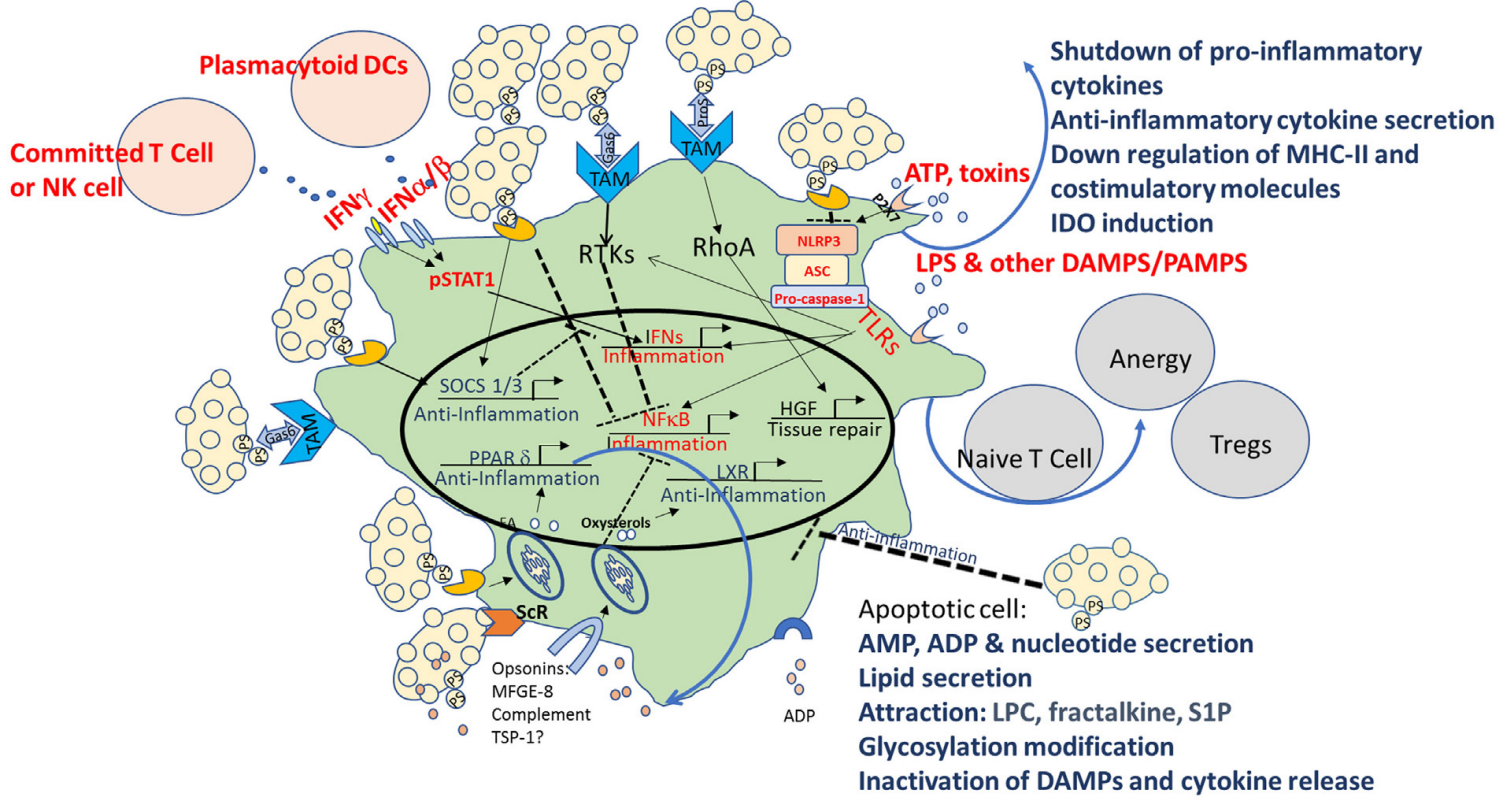
Multiple mechanisms of immune modulation following interaction with apoptotic cells in macrophages and dendritic cells (DCs). Multiple mechanisms are used by apoptotic cells to create an immune homeostatic anti-inflammatory state in macrophages and DCs. In apoptotic cells themselves, in parallel with PtdSer exposure, caspase activation plays a critical role by deactivating potential danger-associated molecular patterns (DAMPs) and by releasing “find me” signals such as adenosine monophosphate (AMP), lysophosphatidylcholine (LPC), fractalkine, and sphingosine-1-phosphate (S1P). Apoptotic cells possess a direct immunosuppressive effect by the release of “calming” agents TGF-β, IL-10, adenosine diphosphate (ADP), thrombospondin-1 (TSP-1), and more. Direct binding to PtdSer receptors (PtsR) and indirect binding to TAM receptors, as well as signaling *via* opsonins/bridging molecules that use additional integrinsor scavenger receptors (ScRs) or complement receptors, act to reprogram the phagocyte, to inhibit toll-like receptors (TLRs), nuclear factor κB (NF-κB), signal transducer and activator of transcription 1 (STAT1), and interferon (IFN) signaling, and to activate liver X receptor (LXR), peroxisome proliferator-activated receptor delta (PPAR-δ), suppressors of cytokine signaling (SOCS) 1/3, and hepatic growth factor (HGF), and to downregulate costimulation and induce induction of indoleamine 2,3 dioxygenase-1 (IDO), that promote tolerogenic phenotype and the induction of T-regulatory (Treg) cell differentiation. The sum of these events leads to downregulation of the inflammatory characteristics of macrophages and DCs, repair, and peripheral tolerance. Pro-inflammatory pattern are marked in red and anti-inflammatory patterns in blue.

Multiple mechanisms are used by apoptotic cells to create an immune homeostatic anti-inflammatory state in macrophages and DCs. As illustrated in Figure 1, these include direct binding to PtdSer and indirect binding to TAM receptors, as well as signaling *via* opsonins/bridging molecules that use additional integrins and ScRs to inhibit TLRs as well as NF-κb, STAT1, and IFN signaling, and to activate LXR, SOCS 1/3, PPAR-δ, and hepatic growth factor (HGF). The sum of these events leads to downregulation of the inflammatory characteristics of macrophages and DCs, repair, and peripheral tolerance. Despite establishment of a pattern recognition effect in the clearance of apoptotic cells, some controversies in the field exist, including the role of complement, the importance of different receptors for PtdSer, the “cross-talk” between different opsonins and receptors, and the specific conditions where “immunogenic” clearance is occurring.

## Author Contributions

DM: prepared the manuscript. UT: Helped prepare the manuscript.

## Conflict of Interest Statement

DM is the founder, chief scientist, and a option holder of Enlivex Ltd. The other co-author declares that the research was conducted in the absence of any commercial or financial relationships that could be construed as a potential conflict of interest.
